# Optimizing Winter‐Type Seasonality Criteria in East Asian Populations: A Machine Learning Approach Using the Seasonal Pattern Assessment Questionnaire

**DOI:** 10.1002/brb3.70834

**Published:** 2025-09-02

**Authors:** Ji Won Yeom, Jung‐Been Lee, Soohyun Park, Chul‐Hyun Cho, Heon‐Jeong Lee

**Affiliations:** ^1^ Department of Psychiatry Korea University College of Medicine Seoul Republic of Korea; ^2^ Chronobiology Institute Korea University Seoul Republic of Korea; ^3^ Division of Computer Science and Engineering Sun Moon University Asan Republic of Korea

**Keywords:** East Asian, machine learning, seasonal affective disorder (SAD), Seasonal Pattern Assessment Questionnaire (SPAQ), winter‐type seasonality

## Abstract

**Background:**

The Seasonal Pattern Assessment Questionnaire (SPAQ) evaluates seasonal variations in mood and behavior. According to Kasper's criteria, individuals meeting diagnostic standards for seasonal affective disorder (SAD) or subsyndromal SAD (S‐SAD) are categorized as winter or summer type based on the month they “feel worst.” However, in East Asian countries with hot, humid summers, relying solely on the “feel worst” item may misclassify seasonality. This study aimed to refine Kasper's criteria using machine learning to improve identification of winter‐type seasonality.

**Methods:**

Among 495 participants from a mood disorder cohort, SPAQ data from SAD and S‐SAD cases were clustered using the *K*‐Modes algorithm into winter type and other types. A decision tree algorithm identified winter seasonality with minimal SPAQ items.

**Results:**

Clustering aligned with additional SPAQ items beyond Kasper's criteria. Respondents selecting a winter month or “no particular month” as “feel worst” were classified as winter type if they also chose a winter month for “gain most weight,” “sleep most,” or “socialize least.” Those selecting a summer month as “feel worst” were considered winter type if they marked a winter month for “gain most weight” or “sleep most.”

**Conclusion:**

This study evaluated seasonality in a South Korean early‐onset mood disorder cohort using SPAQ and Kasper's criteria. Incorporating atypical vegetative symptoms and reduced social activity improved winter seasonality classification accuracy. The revised criteria may facilitate more precise identification and management of seasonal symptoms in East Asia.

## Graphical abstract

Using SPAQ data, this study refines the diagnostic criteria for winter‐type seasonal affective disorder (SAD) in East Asian populations through a machine learning approach. By applying *K*‐Modes clustering and decision tree modeling, the new method moves beyond the traditional “feel worst month” criterion, incorporating key seasonal symptoms—such as hypersomnia, weight gain, and social withdrawal—for more accurate diagnosis.

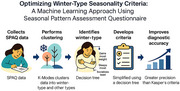

## 1 Introduction

Seasonal affective disorder (SAD) is a subtype of major depressive disorder characterized by recurrent depressive episodes that follow a seasonal pattern, most commonly occurring during the fall and winter months when daylight hours diminish. Initially described systematically by Rosenthal (Rosenthal et al. 1984), SAD is now recognized as a significant mental health issue, affecting millions worldwide. Prevalence rates vary, with estimates ranging from 1% to 10%, depending on geographic location and diagnostic criteria (Magnusson 2000). SAD is associated with symptoms such as lethargy, hypersomnia, overeating, weight gain, and social withdrawal, which, together with its clear seasonal pattern, distinguish it from nonseasonal depression (Rosenthal et al. 1984).

The Seasonal Pattern Assessment Questionnaire (SPAQ) is a widely used self‐report instrument designed to assess seasonal variations in mood and behavior, particularly relevant for SAD and subsyndromal SAD (S‐SAD). Developed by Rosenthal and colleagues in 1987, the SPAQ examines changes across multiple domains—including mood, sleep, social activity, appetite, weight, and energy levels—especially during winter months, when SAD symptoms tend to peak (Rosenthal et al. 1987).

Kasper's criteria, based on the SPAQ, provide a diagnostic framework to categorize individuals by the severity of their seasonal changes (Kasper et al. 1989). This framework includes three main groups: those with SAD, those with S‐SAD, and those without a seasonal pattern. Key indicators in this classification are the Global Seasonality Score (GSS) and the individual's reported difficulty in coping with seasonal changes. Kasper's criteria further distinguish individuals by “season type,” categorizing them as winter type if they report feeling worst in December, January, or February, and as summer type if they report feeling worst in June, July, or August.

For winter‐type SAD, Kasper's criteria identify individuals who experience their worst symptoms during winter, accompanied by notable seasonal shifts in mood and behavior. This approach has facilitated standardized SAD diagnoses in clinical and research settings, especially within Western populations, where most SAD research has been conducted. However, the applicability and accuracy of these criteria in non‐Western populations, such as East Asia, have not been thoroughly evaluated.

Applying Kasper's criteria in East Asian populations presents unique challenges due to the region's distinct climatic conditions and cultural context. Unlike the temperate climates of North America and Europe, where SAD research originated, East Asia has hot, humid summers and relatively mild winters. In South Korea, Japan, and China, extreme summer heat and humidity significantly impact mood and behavior, complicating the assessment of seasonal affective patterns via SPAQ and Kasper's criteria (Lee et al. 2006).

A central challenge in applying Kasper's criteria in East Asia lies in the “feel worst” item used to determine seasonality. In Western populations, individuals often report feeling worst during winter, corresponding to reduced sunlight exposure. In East Asia, however, the intense summer climate may cause some individuals to report feeling worst during summer, despite experiencing traditional SAD symptoms in winter (Lee et al. 2006). This discrepancy can lead to misclassification, as individuals meeting the criteria for winter‐type SAD might not be identified due to reporting worse feelings in summer.

These challenges underscore the need for a tailored approach to diagnosing SAD in East Asia. Revising the criteria to better capture winter‐type seasonality, accounting for unique regional experiences and environmental factors, could enhance diagnostic accuracy and improve treatment outcomes. Given the distinct climatic and cultural factors in East Asia, revisiting and potentially revising the criteria for assessing winter‐type SAD in this population is necessary to accurately identify individuals with winter‐type SAD, even where summer months pose additional challenges.

Machine learning offers a promising approach to enhance the diagnostic process by identifying data patterns that might elude traditional methods. Applying machine learning techniques to analyze SPAQ responses could lead to more refined criteria that align better with the experiences of individuals in East Asia. This approach could improve the accuracy of SAD diagnoses, ensuring that those in need of treatment are correctly identified.

### 1.1 Aims of the Study

This study aims to refine criteria for identifying winter‐type SAD globally, including East Asian populations, through the use of machine learning techniques. Specifically, we seek to reassess traditional Kasper's criteria by incorporating additional factors relevant to winter depression, such as atypical vegetative symptoms and changes in social activity. By developing diagnostic criteria that extend beyond Western contexts, this study strives to improve SAD identification and management across diverse global populations, thereby enhancing mental health outcomes.

## 2 Materials and Methods

### 2.1 Participants and Data Collection

This study was conducted as part of the Mood Disorder Cohort Research Consortium (MDCRC) study, a prospective observational cohort study carried out from July 2015 to April 2019 (Cho et al. 2017). The primary aim of the MDCRC study was to examine longitudinally the characteristics of early‐onset mood disorders in South Korea, as previously reported (Cho et al. 2017, 2024; Kim et al. 2024; Yeom et al. 2021; Seo et al. 2022; Lee et al. 2023). Inclusion criteria included individuals under 35 years of age who had received treatment for major mood disorders, such as major depressive disorder (MDD), bipolar disorder type 1 (BDI), or bipolar disorder type 2 (BDII), for less than two years. Individuals under 25 years with a diagnosis of mood disorders were also included. Exclusion criteria included individuals with intellectual disabilities, organic brain injury, or difficulties understanding the Korean language. The study was approved by the Institutional Review Board of Korea University Anam Hospital (IRB No. 2015AN0239) and adhered to the principles outlined in the Declaration of Helsinki.

Diagnostic interviews were conducted by trained psychiatrists using the Korean version of the Mini‐International Neuropsychiatric Interview (MINI) (Sheehan et al. 1998). Initially, 495 participants diagnosed with mood disorders were recruited.

The final cohort consisted of 480 participants who were followed up every 12 weeks from baseline assessment. Follow‐up duration varied among participants due to sample attrition. Prior to enrollment, all participants completed a comprehensive informed consent process, including a detailed overview of the study.

### 2.2 Seasonality Assessment

The SPAQ (Rosenthal et al. 1987) was used, along with questions related to sociodemographic characteristics. In this cohort, the SPAQ was administered every 3 months during follow‐up; however, for this analysis, only baseline SPAQ assessments were utilized. SPAQ Question 2 calculates the GSS, derived by summing scores from six domains—sleep length, social activity, mood, weight, appetite, and energy level—each reflecting the degree of seasonal change, with a total possible score ranging from 0 to 24. SPAQ Question 3 assesses the overall seasonal impairment score, categorizing responses as none (0), mild (1), moderate (2), marked (3), severe (4), and disabling (5), indicating the severity of seasonal change impacts. Kasper et al.'s criteria were used to diagnose SAD and S‐SAD (Kasper et al. 1989). According to Kasper's criteria, a GSS above 11 with a difficulty score over 2 qualifies as SAD, while a GSS of 9 or 10 with a difficulty score over 1, or a GSS over 11 with a difficulty score of 1 or lower, qualifies as S‐SAD. Of the 495 participants recruited in the MDCRC study, 96 met the SPAQ criteria for SAD or S‐SAD and were included in the final analysis.

### 2.3 Seasonal Subtypes Assessment

To identify seasonal subtypes of SAD and S‐SAD, we used SPAQ Question 1, which includes 10 items addressing extreme values in five dimensions of monthly mood and behavioral fluctuations. Kasper's diagnostic criteria defined seasonal subtype patterns based on the single item “feel worst.” To broaden our analysis, we included additional questions: “gain most weight,” “sleep most,” and “socialize least.” We excluded items such as “feel best,” “lose most weight,” “sleep least,” and “socialize most,” as they represent opposite counterparts to the included questions. In addition, “eat most” was excluded due to its direct cause–effect relationship with “gain most weight.” Ultimately, four questions were selected to cluster participants into seasonal subtypes.

Responses were recorded for each month, with months grouped into four seasons to identify seasonal trends: spring, summer, autumn, and winter. To align with Korea's climate, we redefined seasons as follows: summer from May to September, winter from November to March, spring as April, and autumn as October. For example, if a participant reported “January” as the worst mood month, January was classified as “winter” for clustering purposes, while “August” was classified as “summer.”

### 2.4 Clustering for Identifying Seasonal Characteristics

Clustering techniques commonly applied in machine learning were used to analyze SPAQ response data. Specifically, we employed the *K*‐Modes clustering algorithm (Huang 1997), an extension of *K*‐Means suited to categorical data. While *K*‐Means updates cluster centroids using the mean, *K*‐Modes uses the mode, making it more effective for categorical variables like the four seasons in this study. The algorithm minimizes dissimilarity between data points and their cluster centroids using a simple matching dissimilarity measure.

**FIGURE 1:**
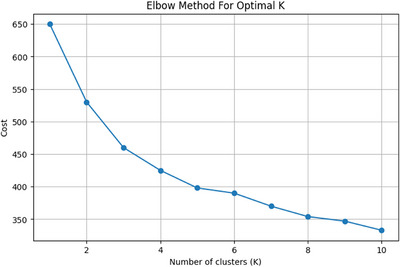
Elbow plot for determining the optimal number of clusters (*K*) in *K*‐Modes clustering. The Elbow method plot showing clustering cost versus the number of clusters (*K*) for the *K*‐Modes algorithm. The point of inflection at *K* = 2 suggests the optimal number of clusters.

Clustering aimed to segment the population into groups with individuals sharing greater similarity within each group than with those in other groups. To justify the choice of the number of clusters in the *K*‐Modes clustering algorithm, we applied the Elbow method by varying the number of clusters (*K*) from 1 to 10. For each *K*, the clustering cost (i.e., the sum of dissimilarities within clusters) was computed. As shown in Figure 1, the cost sharply decreased from *K* = 1 to *K* = 2 and then gradually leveled off for higher *K* values, forming an “elbow” at *K* = 2. This suggests that using two clusters balances both model simplicity and explanatory power. This analysis supports the validity of segmenting participants into two distinct seasonal subtypes, reinforcing the appropriateness of using *K* = 2 for subsequent clustering and interpretation of winter type versus other subtypes (spring, summer, fall).

### 2.5 Identifying Optimal SPAQ Criteria Using a Decision Tree

A decision tree, a supervised learning technique developed by Breiman et al. 1984), was subsequently applied to refine the classification based on clustering‐derived seasonal subtypes. Widely used for both classification and regression tasks, the decision tree recursively partitions data based on specific features, resulting in a tree structure where each node represents a feature, each branch a decision rule, and each leaf a class label or outcome.

The decision tree visually represented the classification process for winter type and other seasonal subtypes based on SPAQ responses and seasonal associations. This method facilitated the identification of essential and nonessential questions within the decision‐making framework, allowing the extraction of a refined subset of questions from the original SPAQ for optimal classification.

## 3 Results

### 3.1 SPAQ *K*‐Modes Clustering Results

**FIGURE 2:**
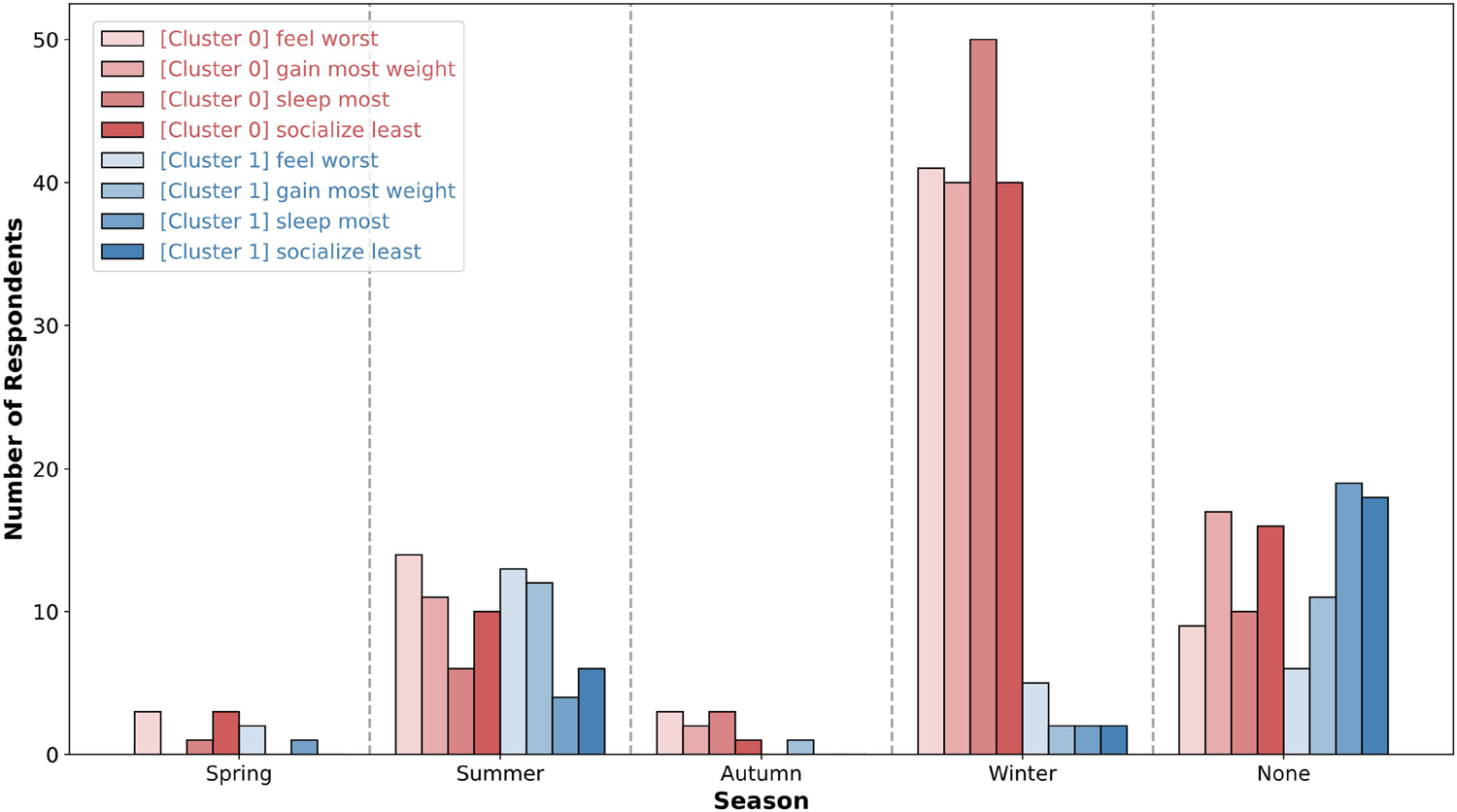
Seasonal preferences across clusters based on SPAQ *K*‐Modes clustering. The red‐toned bars represent the responses from “Cluster 0” while the blue‐toned bars represent the responses from “Cluster 1.”

To distinguish between winter type and other seasonal subtypes, we set the number of clusters at two. Consequently, we clustered 96 patients into two groups, labeled “Cluster 0” and “Cluster 1,” based on their responses to the four questions outlined in Section 3.3. As a result, 70 patients were classified into “Cluster 0” and the remaining 26 patients into “Cluster 1.” To visually examine the characteristics of these clusters, a bar graph was plotted showing the season selected by patients for each question across the two clusters (Figure 2).

As illustrated in Figure 2, “Cluster 0” had a significantly higher number of responses indicating winter across all four questions. For the “feel worst” question, most respondents in “Cluster 0” selected winter, consistent with Kasper's criteria. Similarly, winter was the most frequent response for the “gain most weight” question. In addition, patients in “Cluster 0” tended to report increased sleep (i.e., “sleep most”) and reduced socialization (i.e., “socialize least”) during winter. In contrast, responses from “Cluster 1” rarely indicated winter; most patients in this cluster responded with summer or “none” (indicating no particular month as a consistent extreme). Based on these results, we concluded that “Cluster 0” represents a group with winter seasonality, while “Cluster 1” represents other seasonal subtypes.

### 3.2 Comparison of Seasonal Classification Performance

To assess the consistency between *K*‐Modes clustering and traditional Kasper's criteria, we utilized the Adjusted Rand Index (ARI), a robust statistical measure for evaluating the similarity between two clustering results. ARI is a widely accepted and chance‐adjusted measure used in clustering evaluation (Hubert and Arabie 1985). ARI values range from −1 to 1, where 1 indicates perfect agreement, 0 implies a level of agreement expected by random chance, and negative values indicate worse‐than‐random agreement.

In our study, we compared the seasonal subtypes (winter vs. other) as classified by Kasper's criteria and *K*‐Modes clustering using ARI. The resulting contingency table of classifications is shown in Table 1. Using this table, we calculated ARI of 0.077, indicating low agreement between the two classification methods. This score indicates low agreement between Kasper's criteria and *K*‐Modes clustering. This finding supports the idea that *K*‐Modes clustering captures seasonal subtypes that are not fully reflected by Kasper's criteria. While Kasper's criteria rely on the “feel worst” item to classify SAD types, *K*‐Modes clustering incorporates multiple SPAQ items that reflect the more multidimensional nature of SAD, such as atypical vegetative symptoms (e.g., weight gain and hypersomnia) and reduced social activity. These additional symptoms may be especially relevant in populations like East Asia, where climatic and cultural factors could influence symptom patterns.

**TABLE 1:** Contingency table of seasonal classification by Kasper and *K*‐Modes.

	*K*‐Modes: Other	*K*‐Modes: Winter
Kasper: Other	21	29
Kasper: Winter	5	41

*Note*: Contingency table showing the number of participants classified into “winter” and “other” subtypes by Kasper's criteria and *K*‐Modes clustering. This illustrates the degree of agreement between the two classification methods.

### 3.3 Optimizing SPAQ Criteria Using a Decision Tree

**FIGURE 3:**
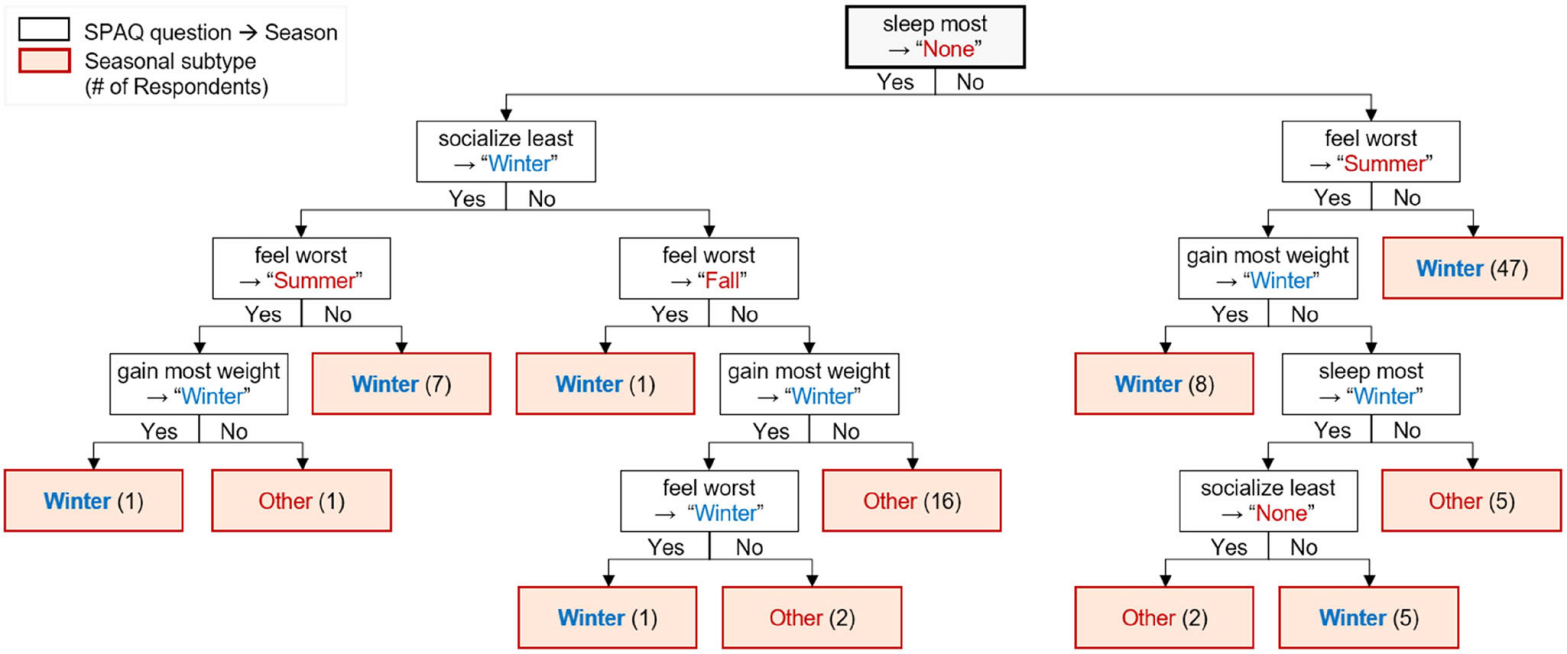
Decision tree for classifying seasonal subtypes based on *K*‐Modes clustering.

In Section 3.2, we showed that *K*‐Modes clustering produced seasonality classifications that differed substantially from those of Kasper's criteria, suggesting that it may capture alternative patterns of seasonal symptoms not identified by the traditional approach. However, the specific contributions of individual SPAQ questions (e.g., “feel worst,” “gain most weight,” “sleep most,” and “socialize least”) to the classification of “Winter” or “Other” seasonal subtypes remained unclear. To address this, we employed a decision tree algorithm, using the seasonal subtypes identified by *K*‐Modes clustering as class labels and the responses from 96 patients to the four SPAQ questions as input variables. The decision tree, depicted in Figure 3, identified the most relevant SPAQ items for distinguishing seasonal subtypes.

For instance, the branch “sleep most → None” indicates that patients who answered “None” to the “sleep most” question were directed along this path. These patients were further classified based on responses such as “socialize least → Winter.” Conversely, if a season other than “None” was selected for “sleep most,” patients followed the branch “feel worst → Summer.” The terminal node labeled “Winter (7)” represents seven patients classified as Winter type, based on the combination of “None” for “sleep most,” “Winter” for “socialize least,” and “Summer” for “feel worst.”

**FIGURE 4:**
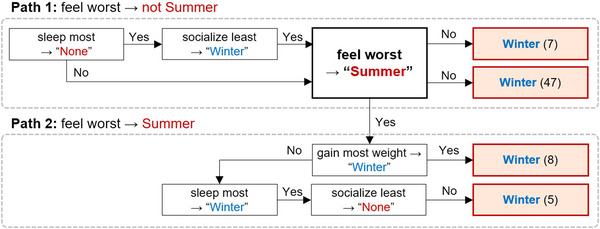
Pruned decision tree for winter‐type classification.

To streamline the decision tree and emphasize Winter‐type classification, we pruned branches leading to the “Other” subtype and those identifying only a single Winter‐type patient (e.g., “Winter (1)”). The resulting pruned decision tree, shown in Figure 4, illustrates the primary decision paths leading to Winter‐type classifications. After pruning, 67 patients were classified as Winter type through four distinct paths.

Path 1 applies to cases where “Summer” was not selected for the “feel worst” question. The first branch in Path 1 includes “Winter” for “socialize least” and “None” for “sleep most,” leading to the classification of seven Winter‐type patients. The second branch captures patients who selected any response other than “None” for “sleep most,” resulting in 47 Winter‐type classifications. Path 2 applies to cases where “Summer” was selected for the “feel worst” question. This path splits into two branches: the third branch, where “Winter” was selected for “gain most weight,” classifying eight patients, and the fourth branch, where “Winter” was selected for “sleep most” and “None” for “socialize least,” identifying five patients.

**FIGURE 5:**
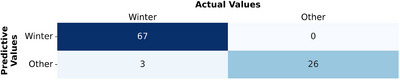
Confusion matrix based on pruned decision tree.

Predicted values (left column of Figure 5) represent model predictions, while actual values (top row) reflect true seasonal classifications. As shown in Figure 5, the model correctly classified 67 patients as “Winter” and 26 as “Other.” Three patients classified as “Other” should have been “Winter,” but no “Other” patients were misclassified as “Winter.” The performance metrics calculated from the confusion matrix are as follows:

- **Accuracy**: The model achieved an accuracy of 96.87%.
- **F1‐score**: The F1‐score of 97.81% reflects a strong balance between precision and recall, effectively identifying Winter‐type patients while minimizing false positives.

To simplify Winter‐type classification, we developed two criteria based on the pruned decision tree. These criteria focus on responses to the “feel worst” question and at least one “Winter” response from the remaining questions, as outlined in Table 2.

**TABLE 2:** Simplified criteria for winter‐type classification.

Criteria	Feel worst	Gain most weight	Sleep most	Socialize least
1	Winter or none	Winter	—	—
		—	Winter	—
		—	—	Winter
2	Summer	Winter	—	—
		—	Winter	—

**Classification criteria**:

- **Criteria 1**: If “feel worst” is “Winter” or “None,” the patient is classified as Winter‐type if any of the following responses is “Winter”: “gain most weight,” “sleep most,” or “socialize least.”
- **Criteria 2**: If “feel worst” is “Summer,” the patient is classified as Winter‐type if either “gain most weight” or “sleep most” is “Winter.”

These simplified criteria significantly enhance the precision of Winter‐type classification compared to Kasper's criteria, which relied solely on the “feel worst” response. Incorporating three additional questions enables a more nuanced and accurate identification of Winter‐type patients.

## 4 Discussion

The primary aim of this study was to propose refined classification criteria for winter‐type SAD that overcome the limitations of Kasper's traditional approach, particularly in East Asian populations. By utilizing machine learning techniques such as *K*‐Modes clustering and decision tree analysis, we sought to derive clinically relevant subtypes based on multidimensional SPAQ data, rather than relying solely on the “feel worst” item.

SAD is a subtype of major depressive disorder characterized by recurrent depressive episodes that occur during specific times of the year. Diagnosing SAD typically involves identifying these recurring depressive episodes within particular seasons, with winter‐type SAD being the most commonly studied due to its association with bipolar disorder‐like features (Forneris et al. 2019). While the cognitive and emotional symptoms of SAD are similar to those of other forms of depression, its vegetative symptoms—notably increased sleep and appetite—are distinct and opposite to those of classic depressive symptoms (Magnusson and Partonen 2005).

These unique symptoms necessitate diagnostic and therapeutic approaches tailored to SAD, differing from those used for unipolar depression. Our study highlights the limitations of traditional Kasper's criteria, which predominantly rely on the month during which individuals report feeling “worst.” These criteria may not adequately capture seasonal patterns in regions with distinct climatic characteristics, such as East Asia, where summers are particularly hot and humid (Lee et al. 2006).

Our findings suggest that using the “feel worst” item alone to determine seasonal type may overlook critical clinical features. For instance, individuals who report feeling worst during summer but experience significant weight gain and increased sleep during winter might be better classified as having winter‐type SAD. Similarly, even if a patient reports feeling worst in winter, atypical vegetative symptoms and reduced social activity must be considered for an accurate diagnosis of winter‐type SAD.

The results of this study underscore the need for a more nuanced approach to diagnosing SAD, particularly in East Asian populations. By applying a machine learning approach, we refined the diagnostic process, addressing the shortcomings of Kasper's criteria (Kasper et al. 1989). Unlike the traditional reliance on a single symptom (e.g., the “feel worst” month), *K*‐Modes clustering takes a more holistic view by considering a range of seasonal symptoms. This approach allows for the identification of clinically meaningful subtypes of SAD that align more closely with the full range of seasonal mood patterns observed in different populations. Specifically, by including symptoms like weight gain, sleep patterns, and social withdrawal, *K*‐Modes clustering reflects a more comprehensive picture of how seasonal changes affect mood and behavior.

This shift toward a more multidimensional diagnostic approach is especially important in non‐Western contexts, where cultural and environmental differences may influence the expression of SAD symptoms. For instance, in East Asia, where summers are long and humid, individuals may report feeling worst in summer despite experiencing the typical winter depressive symptoms associated with SAD. *K*‐Modes clustering is better equipped to capture these complex symptom patterns, providing a more accurate and relevant classification of SAD subtypes.

Despite its strengths, this study has several limitations. First, the relatively small sample size necessitates caution when generalizing the findings to larger populations. Second, the study population primarily consisted of individuals with early‐onset mood disorders, which may not fully represent the general population with SAD. Third, the lack of cross‐cultural evaluations underscores the need for further validation of these findings in diverse regions with varying climatic conditions.

Moreover, while this study effectively identified winter‐type seasonality in the absence of a “gold standard” diagnostic tool, the clustering of SPAQ responses could potentially overestimate winter‐type SAD due to factors such as climate change, ambiguous seasonal boundaries, and, most importantly, the lack of comparison to SADs diagnosed through structured clinical interviews. The gold standard for diagnosing mood disorders with a seasonal pattern entails the use of structured clinical interviews based on DSM criteria. Although the SPAQ is a widely used screening tool for SAD with a good specificity of 94%, its sensitivity has been reported to be only 44% in one study (Mersch et al. 2004). In addition, our exclusion of spring and fall as distinct seasons warrants further exploration to assess the broader clinical applicability of these findings.

Finally, this study examined SADs as a spectrum, including both SAD and S‐SAD. While this inclusive approach provides valuable insights, it may limit the specificity of the findings for SAD alone. Future research should delve deeper into this distinction to enhance diagnostic precision. Environmental factors, such as long and short days, hot and cold weather, sunny and dry days, and pollen count, are also known to affect SAD patients more than non‐SAD individuals (Mersch et al. 2004). How climatological conditions influence seasonality in mood disorder patients warrants further investigation.

The findings of this study present opportunities for future research aimed at refining diagnostic criteria and improving interventions for SAD across diverse populations. One promising avenue involves validating the modified criteria in larger, more demographically diverse samples, including other East Asian and non‐Western populations, to enhance the generalizability of the results. Longitudinal studies that track the stability of SAD symptomatology across multiple seasons would also ensure the robustness of the findings in various climates and environmental conditions.

In addition, as machine learning techniques evolve, further research could explore the use of more advanced models, such as ensemble methods or neural networks, to refine seasonal classification accuracy. Investigating the role of digital phenotyping data—such as wearable device metrics or passive smartphone data—may provide deeper insights into seasonal mood variability and address missing data challenges often encountered in digital phenotyping studies.

Lastly, clinical trials assessing treatment efficacy in patients diagnosed using these refined criteria are essential. These trials could compare intervention outcomes between patients diagnosed with traditional versus modified criteria, providing critical insights into the clinical utility of incorporating atypical vegetative symptoms and social withdrawal into SAD diagnosis.

## 5 Conclusion

In conclusion, this study aimed to move beyond the limitations of traditional SAD classification criteria by introducing novel, data‐driven criteria for identifying winter‐type seasonality. Through unsupervised clustering and decision tree modeling based on SPAQ responses, we demonstrated that a more nuanced classification, incorporating atypical vegetative symptoms and social withdrawal, can enhance diagnostic accuracy in East Asian populations. These refined criteria are particularly valuable in regions where climatic features such as hot and humid summers may obscure classic winter‐depression patterns captured by traditional approaches. More specifically, the proposed criteria can be applied within the DSM‐5 diagnostic framework as a screening tool to determine: (1) whether there is a seasonal pattern in depressive symptoms and (2) if so, which season is associated with greater vulnerability to depressive symptoms. These criteria may help clinicians more accurately assess seasonality in patients with mood disorders and proactively manage them in anticipation of depressive symptoms during specific seasons.

While the present study focused specifically on winter‐type SAD and S‐SAD, and did not explore other seasonal subtypes such as summer‐type or nonseasonal mood variations, the findings provide a meaningful contribution to the nuanced classification of seasonal mood disorders. As such, the proposed criteria are particularly applicable to winter‐type presentations in relevant cultural and climatic contexts, though future research should be conducted to validate their broader applicability to other SAD subtypes.

Overall, our findings support the feasibility and clinical relevance of adopting multidimensional classification strategies for SAD, particularly for populations underserved by Western‐centric diagnostic tools. The current work lays the foundation for a more culturally sensitive and symptom‐informed diagnostic approach to winter‐type seasonal mood disorders.

## Author Contributions

**Ji Won Yeom**: writing – review and editing, writing – original draft, investigation, formal analysis, data curation, conceptualization. **Jung‐Been Lee**: writing – review and editing, writing – original draft, investigation, formal analysis, data curation, conceptualization. **Soohyun Park**: formal analysis, data curation. **Chul‐Hyun Cho**: writing – review and editing, investigation, conceptualization. **Heon‐Jeong Lee**: writing – review and editing, writing – original draft, investigation, formal analysis, supervision, funding acquisition, conceptualization.

## Conflicts of Interest

The authors declare no conflicts of interest.

## Peer Review

The peer review history for this article is available at https://publons.com/publon/10.1002/brb3.70834.

## Data Availability

The data that support the findings of this study are available on request from the corresponding author. The data are not publicly available due to privacy or ethical restrictions.
